# Ultra-Precision Turning of Ferrous and Non-Ferrous Material by Sapphire Tool

**DOI:** 10.3390/mi17060641

**Published:** 2026-05-22

**Authors:** Chung Chi Chiu, Yintian Xing, Wai Sze Yip, Suet To

**Affiliations:** 1 State Key Laboratory of Ultra-Precision Machining Technology, Department of Industrial and Systems Engineering, The Hong Kong Polytechnic University, Hong Kong 999077, China; 19093616r@connect.polyu.hk (C.C.C.); lenny.ws.yip@polyu.edu.hk (W.S.Y.); 2 College of Mechatronics and Control Engineering, Shenzhen University, Shenzhen 518060, China; 3 The Hong Kong Polytechnic University Shenzhen Research Institute, Nanshan District, Shenzhen 518000, China

**Keywords:** sapphire, ultra-precision machining, ferrous material, stainless steel

## Abstract

Ultra-precision machining of ferrous alloys remains challenging because conventional diamond tools suffer severe thermochemical wear, whereas ultrasonic vibration-assisted cutting requires complex and costly equipment. This study investigates single-crystal sapphire as an alternative cutting-tool material for ultra-precision machining of both non-ferrous and ferrous metals. A sapphire tool was fabricated from a polished wafer, laser-shaped into an equilateral triangular insert, vacuum-brazed onto a tungsten carbide carrier, and finished by ultra-fine grinding to yield a well-defined cutting edge. Ultra-precision turning experiments were conducted on copper and 420 stainless steel using a Moore Nanotech 350FG lathe, and the performance of the sapphire tool was benchmarked against conventional diamond (copper) and cubic boron nitride (CBN) tools (stainless steel) under comparable cutting conditions. Surface roughness (Ra) and topography were characterized using an optical surface profiler, while scanning electron microscopy and atomic force microscopy were employed to assess tool wear and cutting-edge geometry. The sapphire tool produced mirror-like surfaces with average surface roughness (Ra) values of 6.4 nm on copper and 39.1 nm on 420 stainless steel, compared with 1.3 nm for diamond on copper and 92.9 nm for CBN on stainless steel. Across both materials, sapphire generated regular, stable tool marks and exhibited minimal wear, with no catastrophic edge degradation or clear evidence of severe chemical interaction with the steel workpiece. These results demonstrate that sapphire is a viable tool material for extending diamond turning-level surface quality to stainless steel without ultrasonic assistance.

## 1. Introduction

Ultra-precision machining has become a cornerstone technology in modern manufacturing of high-accuracy components, where nanometer-level surface finish and dimensional precision are required [1,2]. This advanced technique has been extensively employed in the processing of both metallic and non-metallic materials, including aluminum, copper, and polymethyl methacrylate (PMMA), to satisfy the stringent requirements of optical, electronic, and precision engineering industries [3,4]. Despite these successes, the demand for ultra-precision machining of steels has continued to grow, particularly in applications where wear resistance, strength, and structural integrity are essential [5,6]. 

However, achieving ultra-precision machining of steel remains a substantial challenge due to the incompatibility of conventional diamond tools with ferrous materials [7,8].

The primary difficulty arises from the severe chemical wear experienced by diamond tools during steel cutting. Under such conditions, the cutting zone experiences extremely high temperatures, which promote strong chemical interactions between the iron in the steel and carbon atoms in the diamond lattice [9]. This reaction results in graphitization and rapid deterioration of the cutting edge, significantly reducing tool life and machining quality [10,11,12]. Consequently, the study of alternative tool materials and advanced approaches for the machining of steel with ultra-precision remains an important and active area of research in precision manufacturing [13]. To address the persistent challenge of diamond-tool wear and poor surface integrity in machining steel, researchers have explored a variety of techniques [11]. Early studies attempted to machine carbon steel using diamond tools in a carbon-saturated or controlled atmosphere. The rationale behind this method was to inhibit the diffusion of carbon atoms from the diamond tool into the iron-based workpiece, thereby reducing tool wear caused by thermochemical degradation [14]. However, these approaches provided only marginal improvements, as the diamond tool continued to exhibit rapid wear and unstable cutting performance under conventional conditions [15].

Other researchers redirected their focus to the use of alternative cutting-tool materials, such as cubic boron nitride (CBN), for precision machining of steel [16,17]. Since CBN exhibits significantly lower chemical reactivity with iron compared to diamond, it was considered a promising substitute [18]. Nevertheless, experimental results showed that the tool life remained limited and the machined surface quality did not meet the stringent requirements for ultra-precision applications [1,14]. These limitations prompted researchers to seek additional process modifications to enhance tool performance and surface finish [11,13]. Ultrasonic vibration-assisted cutting (UVAC) systems were introduced as an innovative solution to mitigate diamond tool wear [1,19]. By superimposing high-frequency vibration onto the cutting tool, this approach reduces the contact time between the diamond and workpiece, thereby suppressing adhesion and thermochemical wear [14]. The UVAC method has been particularly successful for the machining of iron-based materials, enabling optical-grade surfaces on hardened steels under appropriate process conditions [6]. In UVAC, the diamond tool tip is driven in an elliptical motion through single- or dual-frequency excitation, producing a high-frequency intermittent contact between the cutting edge and the workpiece surface [5,19]. As a result, the material removal mechanism transitions from a continuous to an intermittent cutting state, significantly decreasing the instantaneous cutting force and chip thickness [13]. Furthermore, the intermittent cutting behavior of UVAC enhances heat dissipation by allowing cutting fluid or ambient air to penetrate the tool–workpiece interface between successive tool engagements [1,14]. This cooling effect reduces adhesion of the workpiece material and mitigates thermal damage, effectively suppressing the thermochemical wear mechanisms of the diamond tool [11]. Despite these advantages, the implementation of ultrasonic vibration systems imposes practical constraints. The method often restricts achievable cutting speeds, increases system complexity, and elevates machining costs due to the high capital and operational expenses associated with vibration-assisted equipment compared to direct cutting with sapphire or conventional rigid tools [6,13,19].

Meanwhile, sapphire, a crystalline form of aluminum oxide (Al_2_O_3_), exhibits exceptional mechanical and thermal properties that make it an attractive candidate material for ultra-precision cutting tools [20,21]. It scores 9 on the Mohs hardness scale and possesses a Vickers hardness exceeding 2000 HV [20], indicating its superior resistance to plastic deformation. In addition to hardness, sapphire has moderate toughness—approximately 5 MN•m^−3^/^2^—and lacks a cleavage plane, meaning it does not fracture easily under sudden impact or stress. These characteristics collectively make sapphire suitable for use as a durable cutting-tool material capable of withstanding demanding machining conditions [17,21].

In precision machining, the cutting tool must generally be at least four times harder than the workpiece to achieve efficient cutting and prolonged tool life [17]. For comparison, the hardness of pure copper is about 37 HV, and that of 304 stainless steel is around 129 HV. Based on these values, sapphire easily satisfies the hardness requirement, demonstrating significant potential for the machining of a wide range of metallic materials [21]. The performance of cutting tools, however, depends not only on their room-temperature hardness but also on their hot hardness, i.e., their ability to retain hardness at elevated temperatures. During machining, the temperature at the cutting edge can exceed 700 °C, leading to softening or wear of tool materials that lack sufficient thermal stability [16,17]. High-speed steels, for instance, exhibit reduced hardness above 600 °C, limiting their effective cutting speed and material compatibility [16]. In contrast, sapphire retains approximately two-thirds of its original hardness, even at 1200 °C [21,22], maintaining dimensional stability and wear resistance under severe thermal conditions. Another critical advantage of sapphire over diamond tools lies in its chemical inertness toward ferrous materials. Diamond, primarily composed of carbon, reacts with iron at elevated temperatures, leading to graphitization and rapid tool degradation [10,23]. Sapphire, on the other hand, is chemically stable and does not undergo such reactions, making it more suitable for direct cutting of steels [21].

In this study, a sapphire cutting tool is fabricated from a raw sapphire wafer through precision grinding and polishing processes [22]. Each manufacturing stage is discussed in detail. The fabricated tool is then tested in ultra-precision machining experiments on non-ferrous copper and ferrous stainless steel, and the resulting process behavior is compared with data reported for diamond and CBN tools where appropriate [18]. Surface topography and roughness are evaluated using an optical profiling system, which is standard practice in ultra-precision machining research. Wear of the sapphire tool is examined after the experiments. The results indicate that the sapphire tools yields improved surface quality for steel workpieces while significantly reducing machining time compared to ultrasonic vibration cutting. Furthermore, the lower cost of sapphire relative to diamond, coupled with the absence of a need for specialized ultrasonic systems, suggests that sapphire tools may offer a cost-effective and high-performance alternative for ultra-precision machining applications.

## 2. Experimental Investigation

To study the feasibility of sapphire as a cutting-tool material for ultra-precision machining, a sapphire tool was developed, and the developed sapphire tool was used for an ultra-precision machining cutting experiment.

### 2.1. Development of Sapphire Tool

To demonstrate the feasibility of sapphire as a material for ultra-precision machining, a sapphire cutting tool was designed, fabricated, and tested in this study. The starting material was an artificially synthesized single-crystal sapphire disk—50 mm in diameter and 1 mm in thickness. Sapphire was selected due to its exceptional hardness, high thermal stability, and chemical inertness, which make it a promising alternative to conventional tool materials for ultra-precision machining.

Both the upper and lower surfaces of the sapphire disk were first carefully polished to achieve a mirror-like finish, producing an average surface roughness of approximately 2 nm. This high degree of flatness and smoothness facilitated subsequent tool fabrication and contributed to improved surface quality in the workpiece during cutting. After polishing, the tool-head geometry was fabricated from the sapphire substrate. A triangular insert geometry commonly used for precision machining applications was selected for ease of assembly and consistency in edge geometry. Laser cutting technology was employed to define the tool-head profile with high precision. In this study, an equilateral triangular tool head, 3 mm in height and featuring a rounded cutting apex with a radius of 0.4 mm, was produced.

After fabrication of the sapphire tool head, it was joined to a DCMT0702 tungsten carbide base conforming to the ISO 1832:2017 standard. Vacuum silver brazing was selected as the bonding method to ensure a strong and thermally stable joint between the dissimilar materials. Subsequently, fine grinding was carried out on the flank face of the tool to generate a clearance angle of 7°. This secondary process also refined the cutting edge, reducing micro-defects introduced during laser cutting and achieving a sharper and smoother edge suitable for ultra-precision machining. Thus, the resulting sapphire cutting tool thus possessed a 7° clearance angle, a 0.4 mm edge radius, and a DCMT0702-compatible base and was ready for cutting experiments.

### 2.2. Experimental Setup

The workpiece materials investigated in this study were a non-ferrous material, i.e., copper, and a ferrous material, i.e., 420 stainless steel. These materials were selected to represent a range of machinability conditions relevant to ultra-precision machining applications. Copper was included as a benchmark material for evaluation of cutting performance, as it is well documented to be compatible with diamond tools and, thus, provides a reference for comparison of with the cutting performance of sapphire tools. Therefore, a diamond tool was used for comparison with the sapphire tool in copper cutting. In contrast, 420 stainless steel is commonly used for precision optical mold inserts and cannot be directly machined with diamond tools due to chemical affinity and tool-wear issues arising from the interaction between iron and carbon. Therefore, this research aimed to assess whether sapphire tools can successfully machine 420 stainless steel while achieving optical-grade or ultra-precision-compatible surfaces. The detailed chemical composition of the 420 stainless steel used in this study is listed in Table 1. For comparison, a cubic boron nitride (CBN) tool was used as a reference standard to benchmark the cutting performance of the sapphire tool in 420 stainless-steel machining.

**Figure 1:**
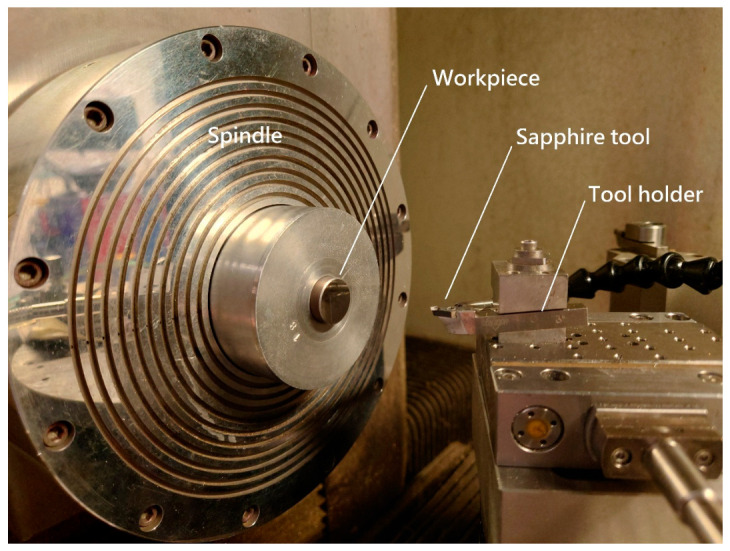
Detail setup of the sapphire-tool cutting experiment.

All cutting tests were conducted using an ultra-precision lathe 350FG from Moore Nanotech, Swanzey, NH, USA. The experimental setup is shown in Figure 1. Each workpiece specimen, 18 mm in diameter, was mounted onto a precision fixture and secured on the machine spindle. The sapphire tool was held in a standard single-point tool holder and mounted on the lathe tool post. The machining procedure consisted of an initial rough-cutting stage to ensure uniformity and flatness of the workpiece surface, followed by a single final finishing cut. The self-developed sapphire tool, the reference CBN, and diamond tools were operated under identical cutting parameters for each material, as summarized in Table 2. Upon completion of each cutting operation, the machined surfaces were examined using an optical profiling system NexView from Zygo, Middlefield, CT, USA to measure surface roughness and characterize the resulting surface profile. Tool wear was investigated using a Tabletop Microscope TM3000 from Hitachi, Hitachi, Japan by taking SEM images of the tool tips after machining. The profiles of the tool tips before machining were measured using an Atomic Force Microscope XE-70 from Park Systems, Republic of Korea to enable better understanding of the cutting performance.

## 3. Results and Discussion

All the workpieces exhibited mirror-like surfaces after the cutting experiments. A total of 17 surface roughness measurements were taken on each sample, together with surface topography measurements at the edge and at the center of each sample. The average surface roughness values are summarized in Table 3.

The experimental results demonstrate that sapphire tools are capable of producing nanometer-scale surfaces on copper and 420 stainless steel, with average roughness values of 6.4 nm and 39.1 nm, respectively. These values confirm that sapphire provides ultra-precision-grade finishes on both materials and, in particular, extends such performance to steels that are traditionally considered difficult or even unsuitable for direct diamond turning. In the following, the results are discussed material by material, beginning with the Ra values, then elaborating on the corresponding surface topographies and cutting stability, followed by an assessment of sapphire’s overall suitability as a tool material for ultra-precision machining.

### 3.1. Non-Ferrous Material—Copper

For copper, the measured average surface roughness was 1.3 nm for the diamond tool and 6.4 nm for the sapphire tool. The diamond result is fully consistent with the state of the art in single-point diamond turning of ductile optical metals and is sufficient for the most demanding optical applications [24]. The sapphire result is not as good as that of diamond under the same cutting conditions; however, an Ra of 6.4 nm still falls within the requirements of many optical components and precision mechanical parts, indicating that sapphire can meet the surface quality needed for a substantial subset of ultra-precision applications. From the roughness data alone, the experiments show that diamond remains more suitable than sapphire for achieving the very best optical finishes on copper, but sapphire nevertheless delivers a mirror-like surface that is functionally acceptable in many contexts.

**Figure 2:**
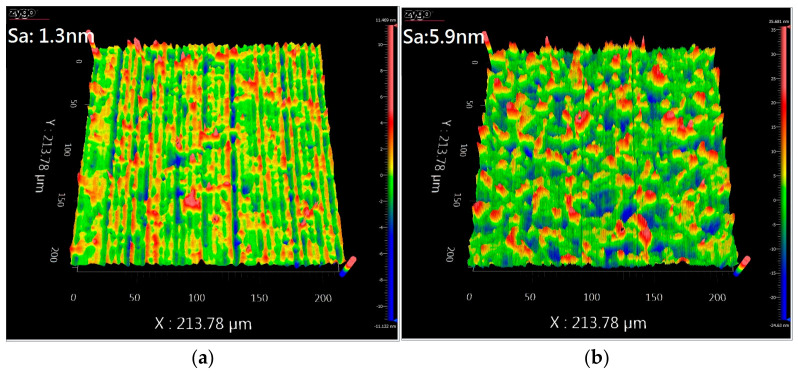
Surface profile of the copper workpiece machined by (**a**) diamond tool and (**b**) sapphire tool.

The surface profiles of the workpieces were also measured, and the results are presented in Figure 2.

The surface topographies on copper support this interpretation. The profiles for both diamond- and sapphire-machined samples exhibit clear and evenly distributed tool marks, as shown in Figure 2a,b. The feed marks follow a regular and repeatable profile, with no evidence of chatter, long-wavelength waviness, or localized damage such as pits or tearing. The diamond-machined surface shows extremely low-amplitude undulations, consistent with the very low Ra of 1.3 nm, whereas the sapphire-machined profile displays slightly larger peak-to-valley amplitudes in line with the 6.4 nm Ra value. Thus, while diamond remains the superior tool for copper in terms of ultimate roughness, sapphire still provides a stable cutting process and ultra-precision-grade surface quality.

**Figure 3:**
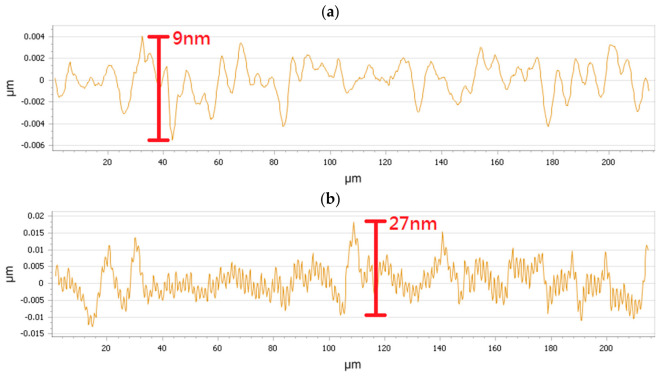
2D surface profiles of the copper workpiece machined by the (**a**) diamond tool and (**b**) sapphire tool, with a peak−to−valley height of (**a**) 9 nm for the diamond tool and (**b**) 27 nm for the sapphire tool is.

Figure 3 compares the 2D surface profiles of copper machined by the diamond and sapphire tools, with peak-to-valley (PtV) values of 9 nm and 27 nm, respectively. Both profiles exhibit regular, periodic feed marks without isolated spikes or deep valleys, indicating a stable kinematic replication of the tool nose rather than sporadic damage events such as pits or tearing. The diamond-turned surface shows very low-amplitude undulations consistent with the measured Ra of 1.3 nm, whereas the sapphire-turned surface presents slightly higher but still well-controlled modulation in the nanometer range, in agreement with its Ra of 6.4 nm. The absence of chatter-induced long-wavelength waviness or random high peaks confirms that the increase in PtV for sapphire reflects a modest degradation in surface smoothness rather than a loss of process stability.

From a surface-generation viewpoint, the PtV difference between 9 nm and 27 nm remains small compared with typical contributions from tool-edge waviness, material springback, and plastic side flow that dominate nanometric topography in ultra-precision diamond turning. Previous studies have shown that such non-ideal effects commonly cause actual PtV values to exceed kinematic predictions by an order of magnitude, even when the underlying cutting process is fully ductile and dynamically stable [25]. The sapphire result is therefore consistent with a slightly larger effective edge radius and enhanced ploughing/side flow relative to diamond but still falls within the regime of controlled ultra-precision cutting. In practical terms, a PtV of 27 nm, combined with Ra = 6.4 nm, lies well inside the nanometric roughness envelope generally associated with ultra-precision optical and precision-engineering surfaces [25].

**Figure 4:**
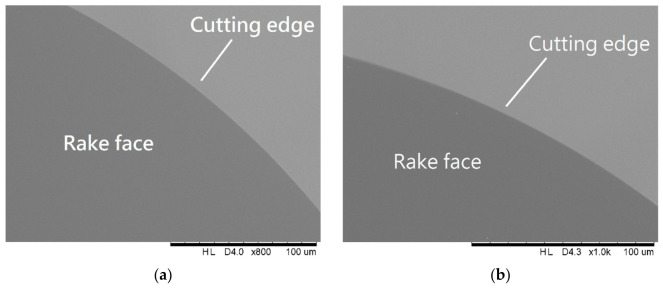
SEM image of the tool tip of (**a**) diamond tool and (**b**) sapphire tool after machining on copper.

SEM analysis of the diamond and sapphire tool tips after ultra-precision turning of copper reveals no discernible wear features, as shown in Figure 4a,b, respectively. Both tools exhibit cutting edges with their sharp geometries intact, devoid of chipping, micro-fractures, abrasive grooves, and built-up edge (BUE) formation typically associated with ductile material machining. The diamond tool preserves its edge sharpness after cutting, consistent with its superior resistance to mechanical deformation against soft copper. Similarly, the sapphire tool shows only negligible surface polishing, without edge rounding or adhesion, indicating wear volumes below the SEM resolution.

This minimal wear underscores sapphire’s suitability for copper cutting in ultra-precision applications from a tool-integrity standpoint. Unlike diamond’s prohibitive cost for high-volume production, sapphire offers comparable durability on non-ferrous metals, with chemical inertness preventing diffusion wear, even at interface temperatures of several hundred degrees Celsius. This positions sapphire as a viable, economical alternative for high-volume copper optics, sustaining mirror finishes over long cutting distances comparable to diamond [26]. Thus, sapphire emerges as a cost-effective alternative for optical copper components, maintaining sub-10 nm finishes under appropriate conditions.

The observed tool conditions directly explain the achieved surface qualities: diamond at 1.3 nm Ra and sapphire at 6.4 nm Ra, both with mirror-like topographies. Diamond’s flawless edge yields ultra-low tool-mark amplitudes (~5 nm PtV), approaching theoretical kinematic limits (Rt ≈ f^2^/8r ≈ 0.6 nm for f = 2 mm/min, r = 0.4 mm). Sapphire’s intact profile produces uniform ~16 nm marks without chatter or defects, with a slight Ra increase reflecting moderated hardness but negligible ploughing due to preserved sharpness [27].

### 3.2. Ferrous Material—420 Stainless Steel

Existing ultra-precision machining studies on steels without ultrasonically assisted cutting generally report surface roughness in the tens-of-nanometers range after turning. For steels such as AISI 420 and 4340, hard turning with CBN tools typically yields Ra values between approximately 40 nm and 200 nm, depending on the cutting parameters and tool condition; achieving an Ra below about 50 nm usually requires optimized geometry and process control [28]. Against this background, the present Ra values obtained by sapphire tools on 420 stainless steel are highly competitive with and, in some cases, clearly better than the conventional CBN and diamond-tool benchmarks reported for these materials in non-ultrasonic ultra-precision processes.

In 420 stainless-steel machining, the average Ra was 92.9 nm for the CBN tool and 39.1 nm for the sapphire tool. CBN is widely regarded as the standard tool material for high-precision turning of steels, and under optimized conditions, it can reach Ra values around or just below 50 nm, but such performance is sensitive to wear and process parameters [28]. In the present experiments, the CBN tool did not achieve ultra-precision roughness, whereas the sapphire tool produced a surface with an Ra below 50 nm, which is already sufficient for some optical and many high-precision engineering applications. This indicates that under the tested conditions, sapphire not only matches but surpasses the performance of CBN on 420 stainless steel. Moreover, the ability to obtain such a finish suggests that no severe chemical reaction occurred between the sapphire tool and the steel, thereby demonstrating the feasibility of directly machining steel with sapphire in the ultra-precision regime.

**Figure 5:**
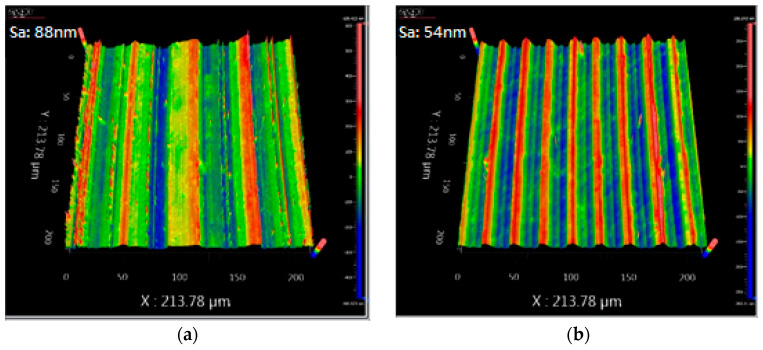
Surface profile of the 420 stainless-steel workpiece machined by (**a**) CBN tool and (**b**) sapphire tool.

The differences are even more pronounced in the surface topography. For the CBN-machined 420 stainless steel, the surface profile in Figure 5a shows tool marks that are not evenly distributed and do not repeat in a regular pattern. Localized irregularities and possible shallow holes or depressions indicate that the cutting process may have been affected by low tool-nose radius quality, progressive CBN tool wear during cutting, vibration, or a combination of these factors, resulting in unstable cutting conditions. In contrast, the sapphire-machined surface in Figure 5b exhibits evenly distributed tool marks with a clearly repeating profile along the feed direction. No holes or severe defects are observed on the surface, and the amplitude of the surface profile remains relatively consistent. This regularity demonstrates that the cutting process with sapphire was stable and that the tool maintained an effective sharpness and geometry throughout the cut. The correspondence between the smooth surface topography and the significantly lower Ra confirms that sapphire improved the surface quality of 420 stainless steel relative to CBN in this study.

**Figure 6:**
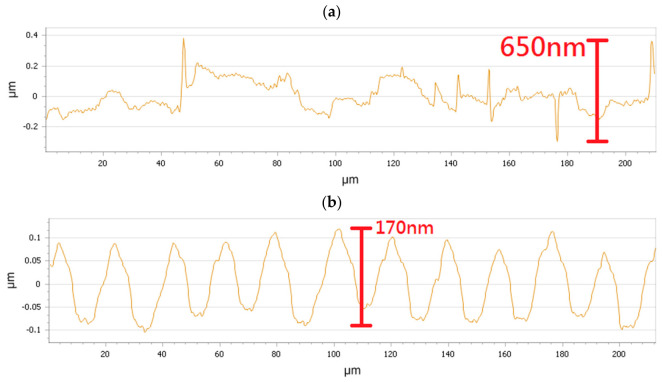
2D surface profiles of the 420 stainless-steel workpiece machined by (**a**) CBN tool and (**b**) sapphire tool, with a peak−to−valley height of (**a**) 0.65 μm for the CBN tool and (**b**) 0.17 μm for the sapphire tool.

Figure 6 presents the 2D surface profiles of 420 stainless-steel workpieces machined by CBN (a) and sapphire (b) tools, revealing stark differences in surface-generation mechanisms. The sapphire-machined surface exhibits clearly repeatable tool patterns with uniform peak-to-valley (PtV) heights of less than 170 nm, demonstrating stable kinematic motion and consistent tool engagement. In contrast, the CBN-machined surface lacks such regularity, showing irregular undulations exceeding 650 nm PtV, indicative of process instability such as chatter or edge degradation. This superior pattern fidelity for sapphire underscores its advantages in ultra-precision machining of ferrous alloys, where predictable surface topography is critical for optical and functional applications.

The sapphire tool’s low PtV aligns with its chemical inertness to iron, preventing affinity wear that disrupts CBN edges and amplifies roughness. Sapphire’s high hot hardness maintains edge integrity, enabling precise replication of feed marks without deflection or vibration amplification, unlike CBN, which suffers from adhesion under similar conditions. Theoretically, in Equation (1), for the experimental feed rate of 16 mm/min (4 µm/rev at 4000 rpm spindle) and a 0.4 mm nose radius, the ideal geometric PtV is

(1) $$R_{t}=\frac{f^{2}}{8r}$$

~5 nm. The measured ~170 nm PtV is substantially higher—attributable to non-kinematic factors prevalent in ultra-precision machining; according to previous research, these factors include tool-edge waviness (~10–50 nm), elastic springback, and ploughing side flow due to a minimum uncut chip thickness of h min ≈ 0.1rn (~40 nm), where rubbing dominates shearing [1,29]. Crystal grain boundaries in polycrystalline steel further contribute ~20–50 nm steps, while micro-vibrations overlay low-amplitude waves [29].

Despite this ~40× deviation from ideal geometry—common in ultra-precision machining, where the actual PtV height is 10–100× the theoretical value due to edge effects and dynamics [1,30]—the sapphire surface remains vastly superior to CBN’s > 650 nm, which is more than 130× from the actual PtV height and is beyond the 10–100× theoretical value. This gap highlights sapphire’s robustness.

**Figure 7:**
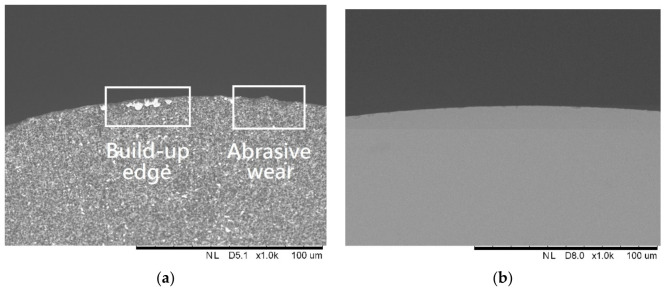
SEM image of the tips of the (**a**) CBN tool and (**b**) sapphire after machining on 420 stainless steel.

Figure 7 displays SEM images of tips of (a) CBN and (b) sapphire tools after machining 420 stainless steel, revealing markedly different wear behaviors. The CBN tool shows a pronounced built-up edge along the rake face, with adherent workpiece material and localized delamination, as well as shallow abrasive grooves on the flank, indicative of combined adhesive and abrasive wear. In contrast, the sapphire tool exhibits only minor polishing of the rake and flank faces, without significant BUE or observable abrasive wear. These observations directly support the surface-roughness results reported earlier, with sapphire achieving an Ra of 39.1 nm, compared with 92.9 nm for CBN, on 420 stainless steel.

In steels, BUE formation is promoted by high cutting temperatures and strong adhesion at the tool–chip interface, which can intermittently alter the effective rake angle, chip flow, and edge geometry, thereby degrading surface integrity [23]. When the BUE periodically breaks off, it tends to leave behind micro-craters on the tool and imprints or tears on the workpiece surface, contributing to the irregular and non-repeating profiles observed for the CBN-cut surface. The abrasive scratches on the CBN tool further indicate interaction with 420 stainless steel, which accelerates edge rounding and increases the minimum uncut chip thickness, pushing the cutting process deeper into the ploughing regime [28,29]. Both mechanisms are consistent with the larger PtV values (>650 nm) and higher Ra values measured on the CBN-machined surface.

In comparison, sapphire’s chemical inertness towards iron and its high hot hardness substantially suppress adhesive transfer and abrasion, resulting in negligible BUE formation and very limited edge degradation [22]. The SEM evidence of only slight polishing suggests that the sapphire tool maintains its original edge radius and geometry throughout the cut, which explains the highly regular, low-amplitude tool marks and the lower PtV and Ra values obtained in Figure 3 and Figure 5. From a tool-life perspective, the reduced wear observed on sapphire implies more stable long-term surface-generation behavior, in contrast with CBN, whose surface quality is highly sensitive to progressive wear and BUE evolution [28]. Thus, these wear mechanisms provide a coherent explanation linking the micro-scale tool conditions to the macro-scale surface roughness and topography trends reported in the present work.

Taken together, the experimental results show that sapphire is capable of serving as a cutting-tool material in ultra-precision machining. It has sufficient hardness and toughness to cut copper and 420 stainless steel under the tested conditions, and post-machining inspection revealed no severe wear or cracks on the sapphire tools. On copper, sapphire produces nanometer-scale finishes suitable for many optical and precision mechanical applications, although diamond remains superior for the most demanding surfaces. On 420 stainless steel, sapphire clearly outperforms CBN in both Ra and surface topography, demonstrating genuinely ultra-precision performance on steel without ultrasonic assistance. The stable, repeatable tool marks and absence of chatter features across both materials confirm that sapphire enables a stable cutting process under good conditions, supporting the conclusion that sapphire is a promising tool material for the extension of ultra-precision machining capabilities to traditionally difficult-to-cut alloys.

### 3.3. Cutting-Edge Radius

**Figure 8:**
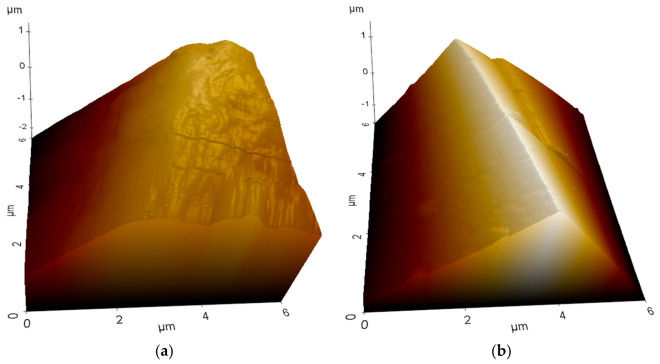
AFM measurement of the tool tip of the (**a**) CBN tool and (**b**) sapphire tool.

Figure 8 shows AFM measurements of the cutting edges of the (a) CBN and (b) sapphire tools, providing quantitative insight into their sharpness and edge quality before machining. The CBN edge appears relatively blunt, with a larger effective cutting-edge radius and a rough, incompletely polished edge zone characterized by pronounced micro-asperities. In contrast, the sapphire tool exhibits a smaller cutting-edge radius and a much smoother edge profile, indicating superior polishing and fewer micro-defects along the cutting edge. These differences in sharpness and edge finish are strongly linked to the surface roughness and process stability observed in the cutting experiments.

In ultra-precision machining, the cutting-edge radius directly influences the minimum uncut chip thickness and the transition between shearing- and ploughing-dominated material removal. Experimental and theoretical studies have shown that when the uncut chip thickness drops below a critical value proportional to the edge radius—typically on the order of 0.05–0.2 times the edge radius—the chip formation process becomes intermittent, with increased elastic recovery, side flow, and frictional rubbing, all of which degrade surface finish [26,30]. A larger and rougher CBN edge therefore raises the minimum chip thickness and intensifies these non-ideal effects, leading to the irregular, high-amplitude surface profiles and higher Ra and PtV values measured on the CBN-machined 420 stainless steel. The poor edge polish also introduces local waviness and micro-notches along the cutting edge, which are directly imprinted onto the workpiece as non-repeating surface features [29].

Conversely, the sharper and smoother sapphire edge reduces the minimum uncut chip thickness and promotes a more continuous shearing mechanism, thereby keeping the discrepancy between theoretical geometric roughness and actual surface roughness within a narrower band [1,30]. Although the measured PtV (~170 nm) remains significantly higher than the ideal geometric prediction (~5 nm), the improved sharpness ensures that the additional roughness arises in a relatively uniform and repeatable manner, producing regular tool marks that are compatible with ultra-precision requirements. This behavior is consistent with broader reviews of surface generation in ultra-precision machining that emphasize edge sharpness and edge waviness as primary determinants of achievable roughness when feed and nose radius are held constant [29]. The AFM results in Figure 8 therefore provide a mechanistic foundation for understanding why sapphire outperformed CBN in terms of both Ra and topography on 420 stainless steel in this study, and they support the conclusion that optimizing tool sharpness is as crucial as selecting the tool material, itself, when extending ultra-precision machining to difficult-to-cut metals.

In this study, the rake face of the sapphire tool was fabricated on the C-plane, and the cutting-edge orientation relative to the crystallographic directions was not systematically optimized. Future work should therefore investigate the influence of different sapphire crystal planes and crystallographic directions on cutting performance, tool wear, and achievable surface quality. Systematic tool-life experiments are also required to quantify the total cutting distance that sapphire tools can sustain under various process conditions and to characterize the corresponding wear mechanisms and patterns in detail. In addition, the present work has focused on copper and 420 stainless steel, but many other difficult-to-cut materials that are traditionally problematic for diamond tools, such as nickel-based superalloys, tungsten, and other refractory metals, remain unexplored. Evaluating the performance of sapphire tools on these materials and assessing their compatibility with different coolants, cutting environments, and machine configurations, will be essential for the full establishment of sapphire as a robust and versatile tool material for next-generation ultra-precision machining.

## 4. Conclusions

This study has demonstrated the feasibility of using single-crystal sapphire as a cutting-tool material for ultra-precision machining of both non-ferrous and ferrous materials. A self-developed sapphire tool was designed, fabricated, and successfully applied in ultra-precision cutting experiments on copper and 420 stainless steel, confirming that sapphire can withstand the mechanical and thermal loads associated with nanometer-scale finishing.

In copper machining, the sapphire tool achieved an average surface roughness of 6.4 nm, compared with 1.3 nm obtained using a diamond tool under similar conditions. While diamond remains superior for the most demanding optical applications, the sapphire tool still produced a mirror-like surface suitable for many optical and precision-engineering components. This indicates that sapphire can act as a practical alternative when slightly higher roughness is acceptable and factors such as tool cost and robustness are of concern.

The advantages of sapphire are most evident in the machining of 420 stainless steel, which is generally regarded as challenging or unsuitable for direct diamond turning. For 420 stainless steel, the sapphire tool produced an average surface roughness of 39.1 nm, which is substantially better than the 92.9 nm achieved with a CBN tool under the same conditions, demonstrating its ability to directly machine stainless steel to an ultra-precision-compatible level. Stable tool marks and the absence of severe surface defects indicate that cutting proceeded under stable and well-controlled conditions, without evidence of catastrophic chemical interaction between the tool and workpiece.

Overall, this work proposes sapphire as a promising tool material for the extension of ultra-precision machining to stainless steels without auxiliary systems such as ultrasonic vibration assistance. Future research should investigate the influence of different sapphire crystal planes and crystallographic orientations on cutting performance, as well as conduct systematic tool-life and wear studies to fully establish sapphire’s industrial viability.

## Figures and Tables

**Table 1:** Chemical composition of 420 stainless steel.

C	Mn	Si	P	S	Cr	Fe
0.15 max	1.00 max	1.00 max	0.04 max	0.03 max	min: 12.0	Remaining

**Table 2:** Experimental cutting parameters of the sapphire tools for different materials.

Workpiece Material	Spindle Speed	Feed Rate	Depth of Cut
Copper	1500 rpm	2 mm/min	2 μm
420 stainless steel	4000 rpm	16 mm/min	6 μm

**Table 3:** Results of cutting experiments with different cutting tools on copper and 420 stainless steel.

	Copper		420 Stainless Steel	
	Average Surface Roughness	Standard Deviation	Average Surface Roughness	Standard Deviation
Diamond tool	1.3 nm	0.09 nm	n/a	n/a
Sapphire tool	6.4 nm	0.67 nm	39.1 nm	14.44 nm
CBN tool	n/a	n/a	92.9 nm	88.22 nm

## Data Availability

The data presented in this study are available on request from the corresponding author.
